# Oxytocin and Estrogen Receptor β in the Brain: An Overview

**DOI:** 10.3389/fendo.2015.00160

**Published:** 2015-10-15

**Authors:** Alexandra Acevedo-Rodriguez, Shaila K. Mani, Robert J. Handa

**Affiliations:** ^1^Department of Neuroscience, Baylor College of Medicine, Houston, TX, USA; ^2^Memory and Brain Research Center, Baylor College of Medicine, Houston, TX, USA; ^3^Department Molecular and Cellular Biology, Baylor College of Medicine, Houston, TX, USA; ^4^Department of Biomedical Sciences, Colorado State University, Fort Collins, CO, USA

**Keywords:** estradiol, oxytocin, hypothalamus, pituitary, adrenal, behavior, anxiety

## Abstract

Oxytocin (OT) is a neuropeptide synthesized primarily by neurons of the paraventricular and supraoptic nuclei of the hypothalamus. These neurons have axons that project into the posterior pituitary and release OT into the bloodstream to promote labor and lactation; however, OT neurons also project to other brain areas where it plays a role in numerous brain functions. OT binds to the widely expressed OT receptor (OTR), and, in doing so, it regulates homeostatic processes, social recognition, and fear conditioning. In addition to these functions, OT decreases neuroendocrine stress signaling and anxiety-related and depression-like behaviors. Steroid hormones differentially modulate stress responses and alter OTR expression. In particular, estrogen receptor β activation has been found to both reduce anxiety-related behaviors and increase OT peptide transcription, suggesting a role for OT in this estrogen receptor β-mediated anxiolytic effect. Further research is needed to identify modulators of OT signaling and the pathways utilized and to elucidate molecular mechanisms controlling OT expression to allow better therapeutic manipulations of this system in patient populations.

## Introduction

The nonapeptide hormone, oxytocin (OT), has gained widespread attention as a potential therapeutic agent in a myriad of disorders, including autism spectrum disorder, schizophrenia, and addiction (1). OT is produced primarily in the neurons of the hypothalamic paraventricular nucleus (PVN) and supraoptic nucleus [SON (2)], is released into systemic circulation, and plays an important role in lactation, parturition, maternal behavior, and pair-bond formation (3, 4). Additionally, OT is released from the PVN axon terminals that project throughout the brain into regions, such as the hippocampus, striatum, and amygdala (5), and has been implicated in the regulation of memory, stress, and social behaviors (1). In addition to traditional release across a synapse, OT is also released from neuron somas and dendrites and can reach nearby brain regions via volume transmission by diffusing across neural tissue (6). In this review, we focus on the function of OT in the brain and its modulation by estrogens.

## Oxytocin Receptors: Brain Distribution and Function

Oxytocin signals through OT receptors (OTRs), which are G protein-coupled receptors that, upon binding to OT, activate the Gq protein subunit and ultimately excite the cell. Autoradiographic studies have identified OTR expression in several regions of the rat brain, including the olfactory system, basal ganglia, hippocampus, central amygdala, and hypothalamus (7). The generation of a knock-in mouse strain where Venus, a variant of yellow fluorescent protein, is under the regulation of the *Otr* promoter sequence, facilitated the identification of OTR expressing cells in additional brain regions, e.g., the median raphe nucleus and the lateral hypothalamus. This mouse model has been valuable in the identification of the phenotype of the cells expressing OTRs. For instance, OTRs have been found in serotonergic neurons (8), implicating serotonergic involvement in OTs anxiolytic effects in depression and anxiety.

Central OT is important in homeostatic processes, such as thermoregulation (9), food intake (10), and mating (11, 12). OT also plays an important role in maternal behavior. Female rats that received and exhibited high maternal care showed higher levels of OTRs in various limbic regions of the brain, including the bed nucleus of the stria terminalis (BNST), central nucleus of the amygdala (CeA), lateral septum (LS), PVN, and medial preoptic area (MPOA). Additionally, central administration of an OTR antagonist (OTA) completely eliminated the elevated licking and grooming behaviors seen in the high maternal behavior animals, suggesting that OTRs mediate maternal behaviors (13).

Additional insights into the function of OT and the OTR are gained from the examination of genetically engineered mouse models. Female OT knockout (OTKO) mice show normal parturition and maternal behavior but are unable to nurse their pups demonstrating that in the mouse OT is not necessary for maternal behavior or labor but is essential for milk ejection (14). Compared to the normal maternal behavior observed in OTKOs, OTR knockout (OTRKO) mice show deficits in maternal behaviors demonstrated by their longer latency for pup retrieval (15). OT signaling is also implicated in social behavior, and the OTKOs and OTRKOs both showed deficits in social memory. Wild-type animals investigate a novel conspecific for a longer period of time than a familiar animal, whereas OTKOs and OTRKOs show similar investigation times for both novel and familiar animals (15, 16). Although OTR levels remain unaltered in OTKOs (14, 16, 17), OTKOs demonstrated increased OTR sensitivity as measured by increased grooming following central OT administration (17).

Unlike OTKOs, OTRKO males display increased aggression in the resident-intruder task (15). It is possible that this elevated aggressive behavior in the OTRKOs is mediated by a lack of OT signaling in the CeA, since administration of OT into the CeA of male rats decreased aggressive behavior (18). Interestingly, OTKO offspring generated from a homozygous breeding scheme demonstrated an increased aggression phenotype as compared to those bred from heterozygous parents, suggesting that OT from the heterozygous dam can prevent the aggressive phenotype in the OTKO pups (15). Although these changes in behavior may relate to the absence of OT or the OTR, these phenotypic changes could be due to compensatory mechanisms that occur during development to overcome the absence of OT signaling.

Furthermore, selective knockout of OTRs in the LS showed that OT plays a bi-directional role in fear regulation dependent on social context. Animals exposed to a non-fearful conspecific [positive social encounter; (19)] or to social defeat [negative social encounter; (20)] during contextual fear conditioning showed reduced or increased fear, respectively, compared to controls. Intra-LS administration of OTA or of a virally linked Cre-recombinase to knockdown OTR expression prevented the altered fear response mediated by the social stimulus (19, 20). These data demonstrate that the OT/OTR system enhances memory of social interactions, reducing fear after positive and enhancing fear after negative social interactions.

Various factors influence OT signaling. OTRs are largely expressed centrally but their regional localization varies across species. For instance, mice and rats both express OTRs in the ventromedial nucleus of the hypothalamus [VMH (7, 8)], but OTRs are not expressed in this region in rabbits (7). These species-specific differences in localization may account for different responses to OT, for example, mice and rats respond differently to OT administration (21). Additionally, mice and humans show different OTR localization. For example, OTR-Venus immunoreactivity was seen in the mouse hippocampus (8) but, in humans, OTRs were not localized to this area (22). OTR signaling also changes during development in rats with transient developmental patterns displayed postnatally, an adult-like expression pattern seen around postnatal day 21, and increased OTR quantities into adulthood (23). Additionally, OT signaling differs between males and females. Female rats were found to have fewer OTRs in the BNST, VMH, and medial amygdala compared to males (24), and in humans, men and women were found to respond differently to intranasal OT administration (25, 26). These differences between males and females may relate to hormone differences, which alter OT signaling and are discussed in more detail in a later section.

## Oxytocin Regulation of Hypothalamic–Pituitary–Adrenal Axis

Oxytocin release from neurons of the PVN and the presence of OTRs within the PVN suggests the possibility that OT can directly modulate the stress reactive hypothalamic–pituitary–adrenal (HPA) axis. The HPA axis responds to stressors and activates neurons residing in the PVN causing increased synthesis and secretion of corticotropin-releasing factor (CRF). The release of CRF into the hypophyseal portal system enhances synthesis and release of adrenocorticotropic hormone (ACTH) from the anterior pituitary. In turn, ACTH acts on the adrenal cortex to stimulate release of glucocorticoids [cortisol in humans and corticosterone (CORT) in rats and mice]. Increased levels of circulating glucocorticoids can further inhibit HPA axis activity via glucocorticoid and mineralocorticoid receptors in the brain as well as acting upon specific brain sites to modulate behaviors (27).

Oxytocin can putatively impact several sites within the HPA axis. PVN neurons that project to the median eminence release OT into the hypophyseal portal vasculature to stimulate adrenal glucocorticoid release by potentiating the actions of CRF at the anterior pituitary level in a similar fashion to the closely related neuropeptide vasopressin (28). By contrast, OT neurons in the PVN that project into the forebrain and release OT in response to stressors (29) exert anxiolytic actions (5). Intracerebroventricular (ICV) administration of OT decreases not only circulating CORT levels but also ACTH levels following exposure to stressors in rats (30, 31) and mice (32, 33), and central infusion of OT into the PVN inhibits HPA axis reactivity, via modulation of CRF neuronal activity (34). Using the restraint stress paradigm in association with OT administration (ICV), Windle et al. (31) demonstrated the presence of an OT-sensitive forebrain stress circuit involving the dorsal hippocampus, ventrolateral septum, and PVN (31).

Endogenous OT levels are also sufficient to alter HPA axis reactivity. ICV injection of OTA showed elevated ACTH and CORT levels prior to behavioral testing suggesting that endogenous OT levels can suppress HPA axis reactivity (34, 35). Additionally, administration of OTA via retrodialysis into the PVN resulted in increased ACTH and CORT release indicating that endogenous OT can inhibit PVN neurons (35). Female OTKO mice show elevated CORT levels following acute and repeated shaker stress compared to wild-type littermates (33), demonstrating a definitive role for OT in regulating HPA axis reactivity to stress.

Interestingly, OT also promotes social buffering in response to stress, similar to the effect seen with fear (19). Female prairie voles subjected to restraint stress demonstrated an increase in anxiety-like behaviors and CORT levels when recovering alone but not when recovering with a male partner, which also corresponded to an increase in OT release in the PVN of these females. Intra-PVN OT injections reduced CORT and anxiety-related behaviors when animals recovered alone, whereas intra-PVA OTA administration prevented social buffering. These observations suggest that PVN OT signaling is necessary and sufficient for social buffering effects in response to stress in prairie voles (36).

## Oxytocin Regulation of Anxiety and Depressive Behaviors

Oxytocin is strongly implicated in social bond formation and social behavior [for review see Ref. (37)], but may also play a role in psychiatric disorders, such as anxiety and depression. The effect of OT in these disorders may be related to abnormal social behavior, but OT may also independently impact these disorders via regulation of the HPA axis. Dysregulation of the HPA axis and increased response to stressors are commonly seen in anxiety and mood disorders (38). In a clinical study with pediatric and adult participants, cerebrospinal fluid and plasma OT levels were found to be higher in participants that had lower anxiety (39). However, severe anxiety symptoms may be related to over-activation of the OT system as women with elevated OT levels were more likely to report being anxious on a daily basis (40). Reduced nocturnal levels of OT have been reported in depressed individuals; however, numerous studies have also reported no differences compared to healthy controls (41). This variability across studies for anxiety and depression may relate to OT levels corresponding more to personality traits rather than symptoms of depression or anxiety (42). Despite these inconsistencies in data concerning psychiatric disorder OT levels, a recent meta-analysis suggests that OT may be beneficial in the treatment of anxiety and depression (43).

Oxytocin signaling during early development may contribute to later anxiety. Prairie vole pups exposed to a single injection of OT on postnatal day 1 demonstrated an increase in serotonergic axon density in the anterior hypothalamus, cortical amygdala, and VMH but not in the PVN or medial amygdala. Such effects on serotonergic neurons could be a mechanism by which OT affects emotional behaviors, since serotonin is strongly linked to mood, and serotonin dysregulation is seen in depression and anxiety disorders (44).

In adult animals, OT administration reduces anxiety-related behaviors in the elevated plus maze (30, 45, 46) and open field assay (8, 11). OT administration centrally (30), to the medial prefrontal cortex (46), to the CeA (11), and to the PVN (45) was sufficient to reduce anxiety-related behaviors. Chronic central OT administration reduced anxiety in rats bred for high levels of anxiety-related behaviors (47). Further support for the role of OT in reducing anxiety comes from studies of OTKO mice with OTKO females showing increased anxiety-related behaviors compared to their wild-type counterparts (32, 33). The effect of OT was sex dependent as male OTKOs showed reduced anxiety-related behaviors (32, 48).

Oxytocin also reduces measures of depression in the forced swim test (FST) and tail suspension test. In FST, rats treated with an OT analog spent less time immobile and more time swimming and climbing the walls of the chamber than saline-treated animals indicating an antidepressant effect (49). Similarly, ICV OT or OTA administration showed a dose-dependent decrease or increase in immobility in both assays, respectively (50, 51). Interestingly, the antidepressant effect of OT was not blocked by a selective OTA, suggesting that OTs antidepressant effects are not OTR mediated (50).

## Regulation of Oxytocin Function by Steroid Hormones

Steroid hormones are a broad family of hormones that include the estrogens, androgens, progestins, mineralocorticoids, and glucocorticoids. These hormones can readily cross the cell membrane where they bind and activate their respective intracellular receptors. Steroid receptor proteins have DNA and ligand-binding domains, and unliganded steroid receptors are maintained in an inactive state by a complex of chaperone proteins (52). Upon ligand binding, the receptors dimerize and translocate into the nucleus and bind DNA promoters and recruit cofactors and transcription machinery to promote gene transcription (53). Steroid hormones have been found to alter OT signaling. Estrogens can act in a synergistic manner with OT, not only by enhancing its anxiolytic effects (54) but also by increasing OTR levels in the mouse brain (55). In humans, a single dose of estradiol was sufficient to increase plasma OT levels in women (56). Similarly, testosterone alters OTR expression differently depending on brain region (21). Progesterone is important in pregnancy maintenance and *in vitro* studies found that progesterone could inhibit OT binding to the OTR (57). Also, treatment with a synthetic glucocorticoid significantly altered OTR expression in various brain regions, such as the amygdala, BNST, and VMH (58).

Understanding OT regulation by sex steroids is important since anxiety and depressive disorders show a larger gender disparity (38), which may be related to circulating steroid hormone levels. Testosterone has been shown to decrease HPA axis activity (59, 60), whereas estrogens can both increase (60, 61) or decrease (62, 63) HPA axis activity, and these alterations may in part be through modulations of OT activity. The differences in the observed effects of estrogens on behavior and neuroendocrine responses to stress may relate to its differential activity on ERα and ERβ. Activation of ERα can increase HPA axis activity, whereas activation of ERβ has the opposite effect (61, 64). Although ERα-mediated activity modulates OTR transcription, ERβ-mediated activity has been found to alter *Ot* mRNA levels (65, 66). Moreover, androgen modulation of OT appears to be mediated in part by the testosterone metabolite 3β-diol, which activates ERβ to allow binding to the *Ot* promoter to increases *Ot* mRNA (67).

## Estrogen Receptor β and Oxytocin Interactions in Regulation HPA Axis and Anxiety-Related Behaviors

Activation of ERβ reduces HPA axis activity, as seen by reductions in ACTH levels and CORT levels, in mice (68) and rats (60, 69, 70) following a stressor. ERβ receptors are expressed widely throughout the brain and often overlap with ERα expression (71), except in the PVN of rats where only ERβ is expressed (72). Interestingly, approximately 85% of OT neurons in the PVN co-express ERβ (72), and activation of ERβ within the PVN, with the ERβ-specific agonist diarylpropionitrile (DPN) or testosterone metabolite 3β-diol, reduces HPA axis activity following restraint stress in rats (61, 73). Treatment with estradiol increases *Ot* mRNA expression in the brains of wild-type mice, but not in ERβ knockout (ERβKO) mice in both males (65) and females (66). This ERβ-mediated increase in *Ot* mRNA was specific to the PVN and not seen in the MPOA, SON (65), medial amygdala, or VMH (66).

The substantial overlap in the distribution of ERβ and OT in the PVN suggests a potential interaction between the two in the regulation of HPA axis activity. As previously discussed, activation of ERβ reduced HPA axis reactivity and anxiety-like behaviors in rats and mice (64, 68, 70). ICV treatment with OTA, however, blocked the ERβ agonist-mediated reduction of anxiety-related behaviors and CORT secretion (70), suggesting interaction between ERβ signaling pathways and OTergic pathways in the control of anxiety-related behaviors and HPA axis reactivity in stress. Currently, the mechanisms involved in the crosstalk between these two pathways are not completely understood.

**Figure 1:**
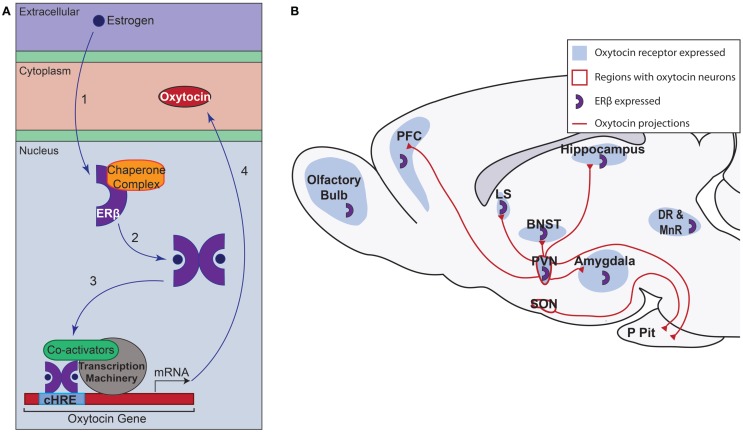
**Overview of estrogen receptor β (ERβ) action in oxytocin neurons of the paraventricular nucleus (PVN) and oxytocin signaling**. **(A)** ERβ signaling in PVN oxytocinergic neurons. (1) Estrogen enters the cell and binds to inactive ERβ. In its inactive form, ERβ is bound to a complex of chaperone proteins. (2) Upon binding its ligand, ERβ dimerizes. (3) The dimerized receptor binds to the composite hormone response element (cHRE) of the oxytocin promoter. Co-activators, such are SRC-1 and CBP, are recruited along with the transcription machinery to promote transcription. (4) Ultimately, the oxytocin peptide is produced. **(B)** Oxytocin signaling in the brain. Oxytocin is produced in neurons of the PVN and supraoptic nucleus (SON). Oxytocin neurons from both regions project to the posterior pituitary (P Pit). In addition to this release, the PVN also sends oxytocin projections throughout the brain (5). ERβ is expressed in approximately 85% of neurons in the rodent PVN but not in the SON (72). This suggests that ERβ could play a role in increasing oxytocin production in regions important in responding to stress and can subsequently influence brain areas that express oxytocin receptors [shown in blue; (8, 13)]. BNST, bed nucleus of the stria terminalis; DR, dorsal raphe nucleus; LS, lateral septum; MnR, median raphe nucleus; PFC, prefrontal cortex.

Recent studies have begun to investigate the complex interaction between ERβ and the *Ot* promoter. Using a mouse hypothalamic cell line expressing ERβ and OT, Sharma et al. (74) demonstrated *Ot* promoter occupancy by ERβ. The *Ot* promoter has a composite hormone response element, which allows for steroid receptor binding and *Ot* gene transcription regulation by ERs and other members of the nuclear receptor family but not the other steroid hormone receptors (75). Treatment of a neuronal cell line with the ERβ agonists, 3β-diol, DPN, or estradiol, elicited increases in *Ot* mRNA levels and *Ot* promoter occupancy (67, 74). In tandem with ERβ occupancy of the *Ot* promoter, cAMP response element-binding protein (CBP) and steroid receptor coactivator (SRC)-1 were found to occupy the *Ot* promoter, leading to increased acetylation of histone H4 in the presence of 3β-diol. Taken together, the data suggest that in the presence of 3β-diol, ERβ binds the *Ot* promoter and recruits ligand-dependent coactivator SRC-1, which binds CBP, and forms a functional complex that acetylates histone H4 to drive *Ot* gene expression (74). The role of ERβ related to OT signaling at the molecular level and its larger role in OT signaling throughout the brain are summarized in Figure 1. Further studies are needed to determine the extent of the binding of ERβ to the *Ot* promoter, the co-activators recruited, and how this interaction modulates HPA axis function *in vivo*.

## Conclusion

Oxytocin has a wide range of roles in the brain and allows interesting and important directions for research. Current data suggest that the OT neurons of the PVN provide the principal OTergic innervation of the forebrain. The function of OT, through OTRs, is regionally specific; however, the localization of OTRs varies across species, age, and sex, so separating the effect of these variables is necessary to determine how animal studies translate to humans. Modulators of the OT system, particularly the steroid hormones, also provide additional regulatory targets since OT modulates HPA axis reactivity and participates in many diverse functions. In particular, ERβ is expressed by many neurons of the PVN, and ERβ activation increases OT synthesis and reduces anxiety and neuroendocrine responses in animals. Hence, such targets may be fruitful directions for future focus.

## Conflict of Interest Statement

The authors declare that the research was conducted in the absence of any commercial or financial relationships that could be construed as a potential conflict of interest.

## Abbreviations

ACTH, adrenocorticotropic hormone; BNST, bed nucleus of the stria terminalis; CBP, cAMP response element-binding protein; CeA, central nucleus of the amygdala; CORT, corticosterone; CRF, corticotropin-releasing factor; DPN, diarylpropionitrile; ERα, estrogen receptor α; ERβ, estrogen receptor β; ERβKO, ERβ knockout; FST, forced swim test; GABA, γ-aminobutyric acid; HPA, hypothalamic–pituitary–adrenal; ICV, intracerebroventricular; LS, lateral septum; MPOA, medial preoptic area; OT, oxytocin; OTA, oxytocin receptor antagonist; OTKO, oxytocin knockout; OTR, oxytocin receptor; OTRKO, oxytocin receptor knockout; PVN, paraventricular nucleus of the hypothalamus; SRC, steroid receptor coactivator; VMH, ventromedial nucleus of the hypothalamus.
